# Meaning of Receiving Artificial Nutritional Support in People in the Postoperative Period of Abdominal Surgery

**DOI:** 10.17533/udea.iee.v38n2e08

**Published:** 2020-07-10

**Authors:** Nieves Fuentes González, Alejandra Fuentes Ramírez

**Affiliations:** 1 Nurse, Masters in Nursing. Assistant professor, Universidad de Boyacá, Tunja, Colombia. Email: nfuentes@uniboyaca.edu.co Universidad de Boyacá Universidad de Boyacá Tunja Colombia nfuentes@uniboyaca.edu.co; 2 Nurse, PhD in Nursing. Universidad de La Sabana. Chía, Colombia. Email: alejandrafuera@unisabana.edu.co Universidad de la Sabana Universidad de La Sabana Chía Colombia alejandrafuera@unisabana.edu.co

**Keywords:** nutritional support, parenteral nutrition, enteral nutrition, qualitative research, postoperative period., apoyo nutricional, nutrición parenteral, nutrición enteral, investigación cualitativa, periodo posoperatorio., apoio nutricional, nutrição parenteral, nutrição enteral, pesquisa qualitativa, período pós-operatório**.**

## Abstract

**Objective.:**

This work sought to describe the meaning of receiving artificial nutritional support in people in the postoperative period of abdominal surgery.

**Methods.:**

This was a qualitative study of grounded theory, following the guidelines by Corbin and Strauss. The information was collected through 26 in-depth interviews with 21 participants, interned in a tier III health care hospital in the city of Tunja, Colombia.

**Results.:**

The study describes four categories, which account for the way in which the person experiences physical, physiological, emotional, and social changes when receiving artificial nutritional support. The categories include stopping eating and becoming artificially fed, decreasing the ability to move to recover movement, experiencing the difficulty of having artificial nutritional support, and reaching the disease to transform life. The data analysis shows that the basic surgical pathology and the artificial nutritional support are sudden events that fragment the daily life of the person. These individuals demand the mobilization of religious, family, and social resources to strengthen the person’s internal and external environment and, thus, achieve the health situation.

**Conclusions.:**

The analysis of the meanings shows how the person reflects and interprets the reality of receiving artificial nutritional support, an event that has implicit physical discomfort, emotional changes, and physical appearance, which are determinants in the behavior and practice of artificial nutrition. However, artificial nutritional support becomes for the person an alternative to live and recover the state of health.

## Resumen

**Objetivo.:**

Describir los significados de recibir soporte nutricional artificial en personas en periodo postoperatorio de cirugía abdominal.

**Métodos.:**

Estudio cualitativo de teoría fundamentada, siguiendo los lineamientos de Corbin y Strauss. La información se recolectó a partir de 26 entrevistas en profundidad a 21 participantes internados en un hospital de tercer nivel de atención en salud de la ciudad de Tunja (Colombia).

**Resultados.:**

El estudio describe cuatro categorías que dan cuenta de la forma en que la persona experimenta cambios físicos, fisiológicos, emocionales y sociales al recibir soporte nutricional artificial en el postoperatorio de cirugía abdominal: dejar de comer y pasar a ser alimentado de manera artificial; disminuyendo la capacidad de moverse hasta recobrar el movimiento; experimentando lo difícil de tener el soporte nutricional artificial, y la enfermedad llega para transformar la vida. La patología quirúrgica de base y el soporte nutrición artificial son eventos súbitos, que fragmentan la vida cotidiana de la persona. En consecuencia, demandan la movilización de recursos religiosos, familiares y sociales para el fortalecimiento del medio interno y externo de la persona y lograr así asumir la situación de salud.

**Conclusión.:**

El análisis de los significados muestra cómo la persona reflexiona e interpreta la realidad de recibir soporte nutricional artificial, evento que tiene implícito molestias físicas, cambios emocionales y en la apariencia física, los cuales determinan en el comportamiento y prácticas de la persona. El soporte nutricional artificial se convierte para la persona en una alternativa para vivir y recuperar el estado de salud.

## Resumo

**Objetivo.:**

Descrever os significados de receber suporte nutricional artificial em pessoas no pós-operatório de cirurgia abdominal.

**Métodos.:**

Estudo qualitativo da teoria fundamentada, seguindo as diretrizes de Corbin e Strauss. As informações foram coletadas através de 26 entrevistas em profundidade com 21 participantes, internados em um hospital de terceiro nível para atendimento de saúde na cidade de Tunja (Colômbia).

**Resultados.:**

O estudo descreve quatro categorias que explicam a maneira pela qual a pessoa experimenta mudanças físicas, fisiológicas, emocionais e sociais, recebendo apoio nutricional artificial no período pós-operatório de cirurgia abdominal: parar de comer e se alimentar artificialmente, reduzindo a capacidade de se mover para recuperar o movimento, enfrentando a dificuldade de ter suporte nutricional artificial e levar a doença a transformar a vida. A patologia cirúrgica básica e o suporte nutricional artificial são eventos repentinos, que fragmentam o cotidiano da pessoa. Eles exigem a mobilização de recursos religiosos, familiares e sociais para fortalecer o ambiente interno e externo da pessoa e, assim, alcançar a situação de saúde.

**Conclusão.:**

A análise dos significados mostra como a pessoa reflete e interpreta a realidade de receber suporte nutricional artificial, um evento que implica desconforto físico implícito, mudanças emocionais e aparência física, determinantes de comportamento e prática diante da nutrição artificial. No entanto, o suporte nutricional artificial torna-se para a pessoa uma alternativa para viver e recuperar o estado de saúde.

## Introduction

The person in the postoperative period of abdominal surgery may be subjected to drastic changes in the way of being fed due to complications associated with the surgical event, co-morbidities, and torpid evolution of the health status, situations that often require management with artificial nutritional support, as the only way to meet the bodily metabolic demands and prevent hospital malnutrition.(1) In the person with surgery, artificial nutritional support has a positive impact upon stopping catabolism activated by the release of stress hormones and inflammatory mediators;(2) inhibition of this process prevents hospital malnutrition, along with its complications, like bedsores, defective healing, increased incidence of the wound dehiscence, fistulizations, postoperative infections, hypoalbuminemia, generalized involvement of the immune system, and bacterial translocation.(3) Besides, it has been documented to reduce hospital stay, readmission rates, mortality,(4,5) care costs(6) and, lastly, relieves feelings of tiredness or fatigue in the person.(7)

Although the clinical results of artificial nutrition are known, it is necessary to know the experience of the person undergoing this situation, given that studies reveal that food not only serves to feed the body, but also has emotional meaning and social value.(8) In addition, it provides the person pleasure and sensations given by the flavor, consistency, and smell of the food(9), which are lost when having artificial nutritional support. Likewise, health situations, like those experienced by the person with abdominal surgery with artificial nutritional support are considered stressful events, which require being described to, as of knowing the meaning, be able to implement interventions aimed at the patient and facilitate the care process, guiding nursing professionals to have a holistic view of the phenomenon under study and contribute new scientific evidence for patient-centered care and on human needs that go beyond the physical body. However, when conducting the scientific literature review regarding the theme, it is scarce globally, which is why the objective of this study was to describe the meaning of receiving artificial nutritional support in individuals in postoperative period of abdominal surgery.

## Methods

This was a qualitative study of grounded theory, following the guidelines by Corbin and Strauss, who denominated it as a theory derived from data collected systematically and analyzed through a research process.(10) It is based on principles of symbolic interactionism. To conduct the study, the researchers did not begin with a preconceived theory, which permits describing and explaining complex phenomena of the individual’s daily experience in natural scenarios; (11) rather, they started from the data obtained from the participants.

Participants were selected through convenience. The principal investigator revised the records of the clinical charts to verify compliance of the inclusion criteria: person hospitalized in postoperative period of abdominal surgery, over 18 years of age and with artificial nutritional support and excluded those diagnosed with cancer, bariatric surgery, and cognitive disorders, as established by the clinical history. Thereafter, the researcher attended the person’s unit to explain in clear and detailed manner the study components. Similarly, doubts emerging during information process were cleared. After explaining the study characteristics, the informant candidate was given reasonable time (between 1 and 2 days) to decide whether or not to participate in the research. During this time lapse, the principal investigator maintained contact and performed care actions, which permitted creating and strengthening empathy ties. Theoretical data was obtained from 26 in-depth interviews to 21 participants; five of them had double inquiry during a second moment to delve into the information collected. The interview began with the question: What do you think about having artificial nutritional support or being fed via a catheter and/or venous catheter? The interviews were recorded and stored in a voice recorder. Moreover, the principal investigator registered the memos in the field diary, which were valuable when performing the data analysis, given that the memos provide density and conceptual integration to the research.(10)

It must be clarified that the interviews were conducted in the units (rooms) of the hospitalization service, which have the necessary elements to provide comfort to the person, a characteristic permitted privacy and facilitated expression and communication between the participant and the researcher; the interviews lasted between 35 and 77 min. Finally, the interviews were transcribed, analyzed, and coded, giving way to the four categories that make up the meanings assigned by the person when receiving artificial nutritional support during the postoperative period of abdominal surgery. Theoretical saturation was considered when the information obtained during the interview did not provide new data during coding.(10)

The study was approved by the ethics committee at Universidad de La Sabana and by the tier II level of care health institution, E.S.E Hospital San Rafael in Tunja, Colombia. The person’s participation in the study was done in free and autonomous manner, by signing the informed consent. Additionally, the study considered that stipulated in Resolution Nº 008430 of 1993, Article 11, which defines minimum-risk research,(12) given that the interview inquires about sensitive elements that affect the participant’s psychological aspects and emotionality. Likewise, the research had minimum impact upon the environment, did not contaminate, according to that stipulated in the 2016 Good Environmental Practices.(13) Regarding methodological rigor, to determine the quality of this study and avoid threats against its validity and reliability, the study used the guidelines proposed by Guba and Lincoln (1985): Credibility, transferability, and confirmability.(14)

## Results

The study recruited 21 individuals with a mean age of 57±15 years. Male gender predominated with 13 individuals; 17 people had parenteral nutritional support; highlighting excelled, appendectomy surgery plus peritonitis in six participants. Analysis of the theoretical data, following the guidelines by Corbin and Strauss, permitted defining the four categories that account for the meaning the person assigns to receiving artificial nutritional support during the postoperative period of abdominal surgery. The following describe each of the categories in function of their properties and dimensions.

### Stopping eating and being artificially fed

*Food does not set well.* The abdominal surgical pathology is mainly manifested with alterations in the intake and tolerance of food. When the individuals eat, they have discomfort, like nausea, vomit, abdominal pain and distension, symptomatology that improves with modifications in diet or with partial or total elimination of the food: *...even when I started eating a little bit, I would tolerate maybe two spoonfuls and nothing more; I could not drink juice until perhaps two hours later, I would drink it and it would start dancing in the gut and that couldn’t be, until it became …* [E02-01, 113-116].

*Associating the health condition with food.* While hospitalized, the individuals reconstruct the events of the disease process, whether through their own deduction or during dialogue with the health professional, which allow them to conclude that food is one of the determinant elements of the health condition, the nutritional state, and prevention of complications during the postoperative period. Furthermore, they recognize in eating cultural, social, and economic representations of the region: *Fatty meat, excess of potatoes, excess of flour, you are told not to eat this or that and it is what you eat most; that is why I am suffering these consequences (patient)* [E06-01, 57-59].

*Needing artificial nutritional support to feed.* Health institutions perform medical and surgical management of the individuals with abdominal pathology. In addition, they conduct nutritional assessment, evaluation of the compromise and functionality of the gastrointestinal tract during the postoperative period, which permits determining the need to set up artificial nutritional support. Artificial nutrition is indicated in this population group to supply the metabolic demands imposed by the disease and to manage and prevent hospital malnutrition. The person upon this new way of being fed feels sadness, depression, and cholera, given that they will no longer eat for an undetermined time and goes on to being fed in artificially, with feeding becoming an involuntary and abnormal act: *The day I was told I was malnourished, that had to get that (points to the parenteral nutrition) that I would get the other, I asked God what I should do. I got somewhat sad …* [E04-01,57-59].

*Identifying differences between being fed with the artificial nutritional support and feeding orally*. The start of the administration of artificial nutritional support permits the individual to identify a series of differences between consuming foods through the natural path (the mouth) and going on to being fed through an artificial path, using medical devices (advanced transpyloric probe and/or central or peripheral catheters). The differences reported by the individual are: feeding without using the mouth, replacement of utensils for devices, and not knowing where the food goes to: *No, it is no longer the same to all these apparatuses I have there (infusion pump), because you grab a spoon and knife (to eat), it is not the same with those apparatuses˝* [E01-01, 128-129].

*Yearning to eat again.* Although the person is receiving artificial nutritional support and the sensation of hunger has disappeared, the desire for eating and perceiving the flavor and consistency of the foods in the mouth remains. Throughout the process of receiving artificial nutritional support, the person yearns to go back to eating like before, through the mouth. *...the only thing is my health and being normal and immediately they will remove the nutrition, the nutritionist and they will give me food through the mouth* [E07-01,132-133].

Diminishing the capacity to move until recovering movement 

*Losing the strength to move.* The individuals assign this meaning, given that prior to starting the process of the abdominal surgical pathology they were active and independent, performed by themselves all the tasks or activities undertaken. Upon the onset of the disease, the nutritional status is compromised due to vomiting, nausea, lack of appetite, pain and alteration in the intestinal transit, symptomatology that interferes with the appropriate contribution and use of the nutrients. Hence, without the adequate caloric-energy intake, the individuals feel discouraged and with loss of strength to walk and perform any type of activity, leading them to being bedridden and being dependent: *At that moment, I felt no strength, I felt no strength, I could not move my arms, I could not, likewise, move my legs - I couldn’t, I did not feel them with strength, I couldn’t …* [E17-01, 51-52].

*Limiting the body’s movement when having the devices.* The supply of the artificial nutritional support requires the installation of invasive devices, which are connected to infusion pumps that deliver exact volumes of nutritional components. When connected to the medical equipment, the person feels that rather than contributing to recover their health, they obstruct and limit the body’s movement, obligating them to seek help from the family or the nursing staff; this makes them feel dependent and useless: *It is always uncomfortable, walking with so many hoses (the enteral nutrition catheter), because I had them in the nose, the hands, they had me from one side to another* [E02-01, 293-294].

*Obtaining the strength to move from the artificial nutritional support.* After the onset of the artificial nutritional support, the individuals identify clearly the positive impact of artificial nutrition on bodily function; they feel more encouraged to go forth, they are capable of moving on their own, this leads them to inferring that they have gained strength and independence: *You get strength from food, it is the key to go forth, if it has to be replaced, if it needs changing, then you have to change it, at least while the health theme improves* [E18-01, 68-70].

### Experiencing the difficulty of having artificial nutritional support

*Modifying the physical appearance when having artificial nutrition support.* When being with the invasive medical devices needed to administer artificial nutritional support, the individuals feel and look differently, perceive that the physical image has modified, the body is not the same as before. Hence, they have to start dealing with physical changes involved: weight loss, looking different due to the devices that invade and hang from the body. Thus, during trajectory of the disease, the individuals yearn to return to being what they were before and that the physical changes are momentary: *My sister took pictures of me this morning, I’ll see if I look at them, to see how I am, because it is always the difference from being well groomed, quite pretty, to being here (hospital), what a difference, one must look different, different with that catheter stuck in there (in the neck)* [E04-01, 246-248].

*Changing one’s self-perception when having artificial nutritional support.* During the process of receiving artificial nutritional support, the person remains connected to medical equipment 24 h per day, a condition that makes them perceive themselves as tied as if they were animals, dependent on a machine and with loss of freedom. Additionally, they assign a serious trait to the clinical condition. This is how artificial nutritional support not only generates changes in the person’s physical aspect, but also permits the construction of a new self image, eminently negative, leading them to feel anguish and at the same time counteract it with the desire to being free at some point during the trajectory of the disease: *Well, I don’t know, because that nutrition (enteral nutrition) is suppose to feed the whole body and you are nourished with that, but tied up there like a lamb* [E11-01, 137-138].

*Feeling physical discomfort with the devices of the artificial nutrition support.* The advanced transpyloric probe causes more physical discomfort than the central venous catheter. The principal discomfort reported by individuals with a probe includes discomfort when breathing and unpleasant feeling in the mouth and throat. However, having any of the two invasive devices is an unpleasant experience for the person, generating sadness, sense of loss of freedom and anguish: *It gives me tremendous anguish (the probe), it does not let you, it does not let you get one wheeze at ease (cough), it makes breathing difficult* [E12-01, 88-89].

*Losing the enjoyment of food.* When substituting the intake of food through the natural path (mouth) and moving to the supply via an artificial path, the individual loses the capacity of deciding the type and amount of food to consume. Likewise, they lose the perception of flavors and consistency of the foods; the habit in eating schedules; now the food is administered continuously 24 h per day: *You do not sense flavor, what flavor will the nutrition have (parenteral nutrition) because if you sensed the flavor, you would say that tastes like, it is okay in salt or it needs some salt. How are you supposed to approve of it?* [E07-01, 97-98].

*Finding the motives to remain with artificial nutritional support.* Accepting artificial nutritional support is not a simple act of approving the installation. The person must be aware that the benefits of the artificial nutrition depend on the permanence and the care taken during the administration. Thus, the person learns to deal with and care for the medical devices that provide the artificial nutrition. *You have to be careful when bathing, sleeping; when sleeping I have to do it on my side and still, otherwise, it can get creased (the advanced probe); one night it got creased (the probe), what a problem it was to reload that hose* [E09-01, 98-100].

### The disease arrives to transform life

*Socializing with people.* The person’s interaction in a health institution produces changes in the family and social dynamics. With respect to the family, the bond with significant others is fragmented, a temporary separation emerges, which must be assumed through the reorganization of the family structure: *…because I can’t be the same there (at home) looking over my children, taking them to school; seeing how they are, what snacks are they taking to school, seeing if they arrived, did they do their homework… (weeping)* [E19-01, 118-120]. Regarding the social part, the person is sharing with the rest in an environment with the specific characteristics of the culture and goes on to occupying an unknown and hostile space due to the rigid norms imposed by health care: *…friendships like those you had at first, with coworkers to go and talk, chat, any person calls you, come over here, come that I don’t know what, how are you doing…* [E16-01, 51-53].

*Being sick.* Although the individuals have gastrointestinal-type manifestations, which indicate that something is occurring in the body, they deny the possibility of thinking about a health problem, until the symptomatology becomes acute, obligating them to seek medical help to treat the health breakdown they are suffering: *You are very reluctant to coming to the; like the saying, nobody likes going to a hospital and to a jail, so you sort of refuse and sometimes you take a pill, drink some herb tea, you sort of skirt the issue of going to the hospital, but the moment comes when you have to, so you must* [E14-01, 147-150].

*Needing help from other people for daily personal care.* Surgical management of the disease, along with unexpected changes in the evolution and treatment of the health condition, like the start of the artificial nutritional support or performance of several surgical interventions, originate in the individual dependence to carry out basic daily life activities, like bathing, dressing, personal care and acre of the skin; activities which are assumed by the family or nursing staff: *…in the physical part it is the same, they also give me foot massages, they rub my back, change my position in bed, all those things, they help me shower* [E19-01, 76-78].

*Reaffirming the belief in God.* After several days of hospital stay, the person lives distressful situations of the medical management, feels that the trajectory of the disease is full of ups and downs; one day they feel vital and positive regarding the situation and the next, they don’t want to continue. However, living the experience of being sick and having artificial nutritional support produces the reaffirmation of the belief in God, who guides the path of all the people involved in the health recovery. The closeness with God leads the person to reflect about life, death, and human transcendence. Moreover, it permits transforming the vision towards the future and offering the tools to accept the health condition positively: *… I know God is there, and I place myself at God’s mercy; I tell God, there is my body, I know you are doing your work with me, at that moment and that is how I believe it, all this process I gave it God and I know He will keep me going ahead from here (hospital)* [E21-01, 29-32].

## Discussion

The following compares the results of this research from the significance with other investigations and theoretical proposals related with the study phenomenon. The first category is *stopping eating and being artificially fed*. The abdominal pathology of surgical management is characterized by gastrointestinal discomfort, counteracted by the person by eating very little or by not eating, measures that generate physical decay and emotional changes in the person. Said findings are related with the qualitative study by Rattray *et al.,*(15) that explored the experiences of patients when starting the feeding process after colorectal surgery. The research revealed that some participants, when experiencing nausea due to the surgical pathology, undertake a series of actions to mitigate the symptom, like, modifying the diet, giving up on foods they normally eat and selecting those they can swallow independent of the enjoyment or avoiding eating food completely. 

Furthermore, the person in the postoperative period of abdominal surgery with compromise of the gastrointestinal tract, malnutrition, or nutritional risk is candidate for management with artificial nutritional support, a clinical situation that generates in the person feelings of sadness by stopping eating and depression due to the health condition, results that coincide with that reported by Hope *et al.(*16) in which patients are depressed due to the health condition and the hospitalization. Lozano-Ballena *et al*.,(17) enhance the scientific evidence when correlating the patient’s nutritional status with abdominal surgery and the short-term surgical results; malnutrition is associated significantly with increased hospital stay, rate of mortality after 30 days, and minor medical complications. 

In this sense, the article on the scientific literature review about clinical malnutrition by Ulíbarri *et al.,*(3) highlights the importance of conducting a nutritional evaluation of the surgical patient, but upon urgent surgical interventions or those that cannot be postponed, nutritional support must be included in the treatment plan for these patients, by following this same research theme and method. 

The second category, *diminishing the capacity to move until recovering movement*; the person in the preoperative and postoperative periods is subjected to prolonged fasting due to the clinical condition of the disease and restrictions imposed by the health condition, circumstances that generate consumption and depletion of the bodily energy reserves. These alterations in the metabolism are perceived by the person with loss of strength to move, weakness and decay, manifestations that when perpetuated lead the individual to becoming dependent on others. The cohort observational study by Sorrel *et al.,(*18) supports the findings of this research. Upon revealing that parenteral nutrition at home affects the person’s quality of life, the aforementioned associated with diminished mobility and psychological problems due to restrictions imposed by the artificial nutrition. Additionally, the medical devices (infusion pump) limit body movement; over time, the individuals learn to move with them until they finally get used. Results consistent with the study by Wong *et al*.,(19) who used the grounded theory methodology to describe the experience of living with parenteral nutrition at home and a stoma. The authors reported two problems experience by participants with parenteral nutrition; the first is the loss of mobility in the home due to the infusion pump and the nutrition bag; the second is the maintenance of the infusions for long periods of time.

In turn, the participants in the present study stated the need for help to perform basic daily life activities due to the loss of energy and strength caused by the disease and the nutritional imbalance. Thus, it i show artificial nutritional support provides the nutritional elements fundamental to recover strength and movement. Tobberup *et al.,(*20) found in a systematic review and meta-analyses, a prospective randomized controlled study that evidenced positive changes in physical performance in the six-minute walk test in patients assigned randomly to receive parenteral nutrition.

The third category, *experiencing the difficulty of having artificial nutritional support*, is related with changes the person perceives regarding the physical appearance, perception of themselves, and loss of satisfaction and pleasure generated by eating by their own means. These results agree with the systematic review of qualitative and quantitative studies on the attitudes and barriers in feeding due to endoscopic gastrostomy, conducted by Jaafar *et al.,(*21) who reported that one of the negative aspects in adapting and incorporating nutrition through probe on the person’s lifestyle is the loss of pleasure for food. For the individual, food becomes a fuel and not an activity of leisure or pleasure. Another difficult experience lived by the individuals when having the devices of artificial nutritional support (central venous catheter and advanced transpyloric probe) is the modification of the physical appearance. These findings agree with the study by Wong *et al.,(*19) which reported that the research participants experienced loss of self image and increased self-conscience, elements that can trigger psychosocial problems.

In the fourth category, *the disease arrives to transform life,* the meaning from the person’s perspective makes the experience lived by receiving artificial nutritional support to permeate all the spheres of the person’s existence. The people in this study state in their testimonies that the family provides them love and aid in personal care activities. Said results agree with the qualitative thematic analysis study by Halliday *et al.,(*22) about the experiences of patients and caregivers of living with nutrition probe in the postoperative period of esophago-gastrectomy and which reported that the family and loved ones are the principal source of support to comply with daily activities.

Agreeing with the reaffirmation of the belief in God, in that reported by Gariella *et al.,*(23) the researchers conducted a cross-sectional pilot study on the importance of spirituality and religiosity in 101 patients with burn injury at various stages of reconstructive surgery, revealing that patients in postoperative place God as first priority in life. Besides, the participants spent more time in prayer and meditation. Balboni and Balboni(24), in a cross-sectional multicentric study, which sought to describe how spirituality operates in the experience of cancer, reported that patients state being moderately religious and spiritual, but religiosity and spirituality increase with the onset of a life-threatening disease. Results that in the religiosity part are similar to that manifested by the individuals in the present study, who referred that surviving the disease and the loss of strength made them reflect about life and death, a situation that got them closer to God.

Finally, it is concluded that the individual upon receiving artificial nutritional support assigns a series of meanings to this life event, which begins with the presence of gastrointestinal symptomatology and culminates with abdominal surgical intervention, an event that potentiates malnutrition or nutritional risk, making it necessary to start artificial nutritional support. Upon said circumstance, the individuals deconstruct the meaning of feeding by their own means and assign a new one: that of being fed to maintain life. In this process, there are changes in the person of a social, emotional, and self-perception nature, to finally make sense of the experience. In addition, it permits health professionals to change their views about the person with artificial nutritional support, who is not a passive receptor or an object of medical treatment, but a person who receives the benefits of artificial nutrition. Hence, the person must be cared from the biological, social, and emotional aspects, thus, the study invites to delve further into the phenomenon.
